# The Corpus Callosum and the Visual Cortex: Plasticity Is a Game for Two

**DOI:** 10.1155/2012/838672

**Published:** 2012-06-21

**Authors:** Marta Pietrasanta, Laura Restani, Matteo Caleo

**Affiliations:** ^1^Laboratory of Neurobiology, Scuola Normale Superiore, Piazza dei Cavalieri 7, 56100 Pisa, Italy; ^2^CNR Neuroscience Institute, Via G. Moruzzi 1, 56124 Pisa, Italy

## Abstract

Throughout life, experience shapes and selects the most appropriate brain functional connectivity to adapt to a changing environment. An ideal system to study experience-dependent plasticity is the visual cortex, because visual experience can be easily manipulated. In this paper, we focus on the role of interhemispheric, transcallosal projections in experience-dependent plasticity of the visual cortex. We review data showing that deprivation of sensory experience can modify the morphology of callosal fibres, thus altering the communication between the two hemispheres. More importantly, manipulation of callosal input activity during an early critical period alters developmental maturation of functional properties in visual cortex and modifies its ability to remodel in response to experience. We also discuss recent data in rat visual cortex, demonstrating that the corpus callosum plays a role in binocularity of cortical neurons and is involved in the plastic shift of eye preference that follows a period of monocular eyelid suture (monocular deprivation) in early age. Thus, experience can modify the fine connectivity of the corpus callosum, and callosal connections represent a major pathway through which experience can mediate functional maturation and plastic rearrangements in the visual cortex.

## 1. Introduction

From playing the piano to riding a bike, interhemispheric communication is a crucial tool that our brain uses to perform a variety of everyday actions, from very simple to complex behaviours. One of the most important pathways through which this communication is achieved is the Corpus Callosum, the major fiber bundle in the brain [1–3]. In humans it contains about 170–190 millions of fibers that interconnect homologous cortical areas in the two hemispheres, as estimated by a pioneer work on human brains by Tomasch in 1954 [4].

A number of experiments and observations suggest that the two hemispheres could inhibit each other via the callosal pathway, to achieve the segregation of lateralized functions such as language or face recognition [3, 5]. However, other experiments suggest that the callosal pathway provides an excitatory input to the opposite hemisphere that enables cortical integration [5, 6].

In vision, our main sensory system, the corpus callosum serves to bind together the separate representations of the two halves of the visual field [2, 7, 8]. Indeed, each hemisphere receives information from the opposite visual hemi-field; thus, the visual world is represented discontinuously in cortical maps, being split between the two hemispheres along the central vertical meridian. One key role of the callosum is to combine these two partial cortical maps of the visual field into a single, coherent representation [9]. Recent electrophysiological data obtained in the ferret confirm that callosal connections integrate the visual field across the vertical midline in a stimulus-specific manner [10].

## 2. Anatomy of Callosal Projections in the Visual Cortex 

Consistently with a role in perceptual binding, callosal connections are strongly concentrated in a zone at the border between areas 17 and 18, corresponding to the representation of the vertical meridian in cats, macaques, and humans [9, 11, 12]. Recent studies have employed diffusion tensor imaging to show evidence for callosal connections in human V1 [13–15]. The callosum links cortical loci that are in retinotopic correspondence [16, 17]. Neuroanatomical tracing in cats shows that clusters of callosal boutons are preferentially distributed in regions representing also the same orientation and not only the same visuotopic location in the opposite hemisphere [18].

In cats, interhemispheric fibres originate from a narrow transition zone between area 17 and 18 [8] and, according to the general rule that retinotopic loci are callosally connected, callosal terminals in the opposite hemisphere are particularly concentrated in this area 17/area 18 border [19–21]. Tracing studies show that callosal axons display 2 or 3 clusters of synaptic boutons in layers 2-3 and the upper part of layer 5 [18].

In rodents, different from cats and primates, the entire extent of the primary visual cortex contains callosal cells [22]. However, their terminals are still particularly concentrated in a quite narrow stripe at the area 17/18 border [23, 24]. Recently, Mizuno et al. [25] have confirmed this findings in mice by labelling callosal axons via in utero electroporation of green fluorescent protein. These experiments have also shown that axonal arborisations of callosal cells are mainly located in layers 1–3 and layer 5 [25, 26].

Callosal cells do not constitute a homogenous population, since they have different morphochemical phenotypes [8, 27, 28]. In cats, the vast majority of callosal neurons are large pyramidal cells, and immunohistochemistry observations and studies using the selective uptake of radiolabeled transmitters have failed to identify GABA containing callosal neurons [29]. Nevertheless, experiments with retrograde transport of horseradish peroxidase injected into border region of the opposite hemisphere report the occasional observation of transcallosal nonpyramidal cells in cats [30]. Also, transiently during early rat development immunocytochemical staining reveals numerous GABA-positive fibres in the callosum, which largely disappear at later stages [31]. In keeping with these results, our group has shown that in juvenile rat visual cortex only 1% of callosally projecting neurons display GABA immunoreactivity [32].

In monkeys, chimpanzees, and humans, callosal axons of distinct size interconnect functionally different cortical areas [33, 34]. The axons originating from each cortical site cover a considerable range of conduction velocities, dispersing in time the action potentials transmitted to the other hemisphere. A wide range of temporal delays might expand the number of neuronal ensembles that transcallosal connectivity can activate [33].

## 3. Physiology of Callosal Connections in the ****Visual Cortex

Electrophysiological observations have shown that callosal inputs can provide both excitation and inhibition to the contralateral side [35, 36]. On the one hand, the removal of the callosal input to the opposite visual cortex (via cooling or GABA injections in one hemisphere) results in a decrease of neuronal responsivity in a fraction of the recorded cells, suggesting a callosal excitatory contribution to these neurons. On the other hand, a subset of neurons show an increase in the response magnitude, compatible with the removal of a callosally driven inhibition [35, 36].

These physiological data showing transcallosal inhibitory and excitatory effects are corroborated by the intra- and extracellular results obtained in cats by different groups showing that callosal fibers mainly evoke a direct excitation of neurons in the opposite hemisphere but can also produce a disynaptic inhibitory postsynaptic potential via a local GABAergic cell [8, 37].

The type of information transmitted by the corpus callosum to the visual cortex has been studied more recently by electrophysiological, optical imaging and psychophysical approaches.

Recordings of local field potentials before, during, and after inactivation by cooling of the opposite hemisphere demonstrated that callosal input modifies visual responses in a complex and stimulus-dependent manner [10]. Specifically, callosal influences more frequently depress the responses elicited through the thalamocortical pathway (indicative of interhemispheric inhibition), but facilitatory events are also observed [10]. This callosal excitation is mainly between neurons tuned to the same orientation, consistent with anatomical evidences of direct monosynaptic connections linking neuronal clusters representing the same orientation in the two sides of the brain [18]. Conversely, transcallosal inhibition is both between iso-oriented and cross-oriented neurons. It is possible that this effect is mediated via local interneurons and spread of GABAergic inhibition across columns of different orientations [10]. 

The callosum also modulates visual response properties, like orientation and direction selectivity across the midline. In particular, in the cat, callosal connections contribute to the strength and specificity of the orientation and directional response in cortical neurons [38]. Cortical domains preferring cardinal contours seem to receive a strong inter-hemispheric input, that is lost after cooling of the contralateral hemisphere [38].

Another key function of the visual callosal connections is to create transhemispheric neuronal assemblies by synchronizing the activity of neurons in the two hemispheres. Indeed, section of the corpus callosum or inactivation of one side substantially impacts functional coupling of the two hemispheres [39–42]. 

Callosal connections also play a role in determining cortical binocularity and in other functions such as depth perception, horizontal disparity tuning, contrast sensitivity, and transfer of adaptation [38, 43–45]. Spatial and temporal characteristics of the visual information transmitted through the callosum are similar to those of a lowpass filter, indeed high spatial and temporal frequencies are attenuated, and callosal neurons have reduced sensitivity to low contrasts [43]. A recent study in human has confirmed the importance of callosal communication in processing high contrasts. Indeed, after rTMS silencing of the left visual cortex the authors found a selective increase, in the opposite hemisphere, of field potentials evoked by high-contrast stimuli [46].

## 4. Development of the Callosum: Role of ****Spontaneous Activity and Visual Experience

The development of the corpus callosum is a slow process that spans many years in humans; the fibers appear at 10-11 weeks of gestation but the maturation continues until myelination is completed during puberty [47]. In cats, the callosum is fully developed between 1 and 3 months of age [48]. In rodents maturation of the callosum is complete just after eye opening (postnatal day 15) [25], but the process of myelination continues into adulthood [49].

Visual callosal axons are initially exuberant, but during development they undergo a phenomenon of partial elimination [50, 51]. During the first two postnatal months in cats, the callosal efferent zones become progressively restricted to their adult locations in visual cortex [51]. Also in the rat parietal cortex, the major factor in the progressive restriction of thecallosalprojection is the withdrawal or degeneration of axon collaterals, rather than the selective death of many of the cells that initially project to the opposite side [52]. Initial exuberance of neuronal connectivity followed by a later phase of axon pruning is a common theme in neural development [53].

Like any other brain structure, the callosal pathway can undergo plastic changes during its early formation and maturation. It has been shown that the development of visual callosal connections is strongly dependent upon neural activity even before eye opening [25, 56]. Neonatal enucleation experiments show that activity is required for the refining of callosal projections. Electron microscopy on sections from enucleated rats shows that eye presence is necessary for the development and/or maintenance of callosal terminals forming multiple synaptic contacts [57]. Eye presence is also important during a window of callosal plasticity (from postnatal day 4 to 6 in rats and mice) to specify callosal maps in a non-NMDA receptor-mediated process [17, 58]. NMDA receptors seem to be required mainly for the initial elaboration of callosal arbor development [17]. In mice, Mizuno et al. [25] explored the role of spontaneous activity in callosal development, showing that a decrease in firing activity of callosal neurons leads to an impaired growth of axon and their arbors. Conversely interfering with firing of callosal target neurons has only a limited effect on the pattern of callosal terminals [25]. During development there has been also demonstrated the presence in the rat cortex of a substantial, but transient population of functional GABAergic transcallosal neurons. These GABAergic neurons are detectable perinatally but do not seem to persist into adulthood and could work as pathfinding or differentiation cues [31]. There are also a few identified molecular determinants responsible for the callosal fate of excitatory projecting neurons in mouse cortex. In the absence of *Fezf2,* a zinc finger transcription factor, cortical neurons adopt the axonal targeting of callosal neurons and their typical strong spike frequency adaptation in response to intracellular current injection. *Fezf2* ^−/−^ neurons also acquire the expression of a known callosal marker, the chromatin remodeling protein Satb2 [59–61]. 

Modulation of visual experience also affects the development of callosal connections. Rearing animals in complete darkness from birth exaggerates the partial elimination of callosal projections, with fewer terminating callosal axons at the area 17/18 border [62, 63]. Similarly, bilateral eyelid suture causes a clear reduction of callosal connections, with a 50% reduction of the total number of callosal neurons [64]. Conversely, monocular enucleation produces an abnormally wide distribution of callosal cells at the 17/18 border. This latter effect is similar to that described in cats reared with convergent or divergent strabismus, or monocular eyelid suture. All these manipulations produce a widespread distribution and exuberant number of callosal terminals [65–68].

There are also data showing that visual experience can influence the functional properties of callosal neurons in adult cats. For example, a study demonstrated that MD in adulthood is able to induce functional changes in visual callosal map, leading to an increase of receptive field size and to a loss of orientation selectivity [69].

## 5. Role of the Callosum in Developmental ****Maturation of the Visual Cortex

The experiments described so far demonstrate that spontaneous activity and sensory experience can modify the fine connectivity of the corpus callosum. The question arises whether there is a role for inter-hemispheric communication in visual cortical development. The visual cortex is immature at the time of eye opening and gradually develops its functional and structural properties during a critical period early in life [70]. During this time window, experience refines a number of visual properties. Among these, an important marker of maturation is the increase of visual acuity, that in rats reaches adult values around postnatal day 35. In parallel with the maturation of acuity, there is a progressive loss of the potential for plasticity in the cortex. This is usually demonstrated by a downregulation of the effect of a period of monocular eyelid suture (monocular deprivation, MD) on eye preference of cortical cells [70, 71]. Many studies have described the role of visual experience in visual cortex maturation [71–73]. Total lack of visual experience by dark rearing, for example, halts maturation of visual acuity and prolongs the period of sensitivity to MD [71, 73]. While the role of visual experience in cortical maturation is well established, our group has recently addressed the specific role of callosal connectivity in functional development of the visual cortex. Specifically, we produced a unilateral, prolonged silencing of activity in the developing rat primary visual cortex by taking advantage of the clostridial enzyme botulinum neurotoxin E (BoNT/E). BoNT/E is a metalloprotease that enters the cytosol of nerve terminals close to the site of delivery and specifically cleaves the synaptic protein SNAP-25 (synaptosomal-associated protein of 25 kDa), causing a prolonged blockade of transmitter release [74–76].

**Figure 1:**
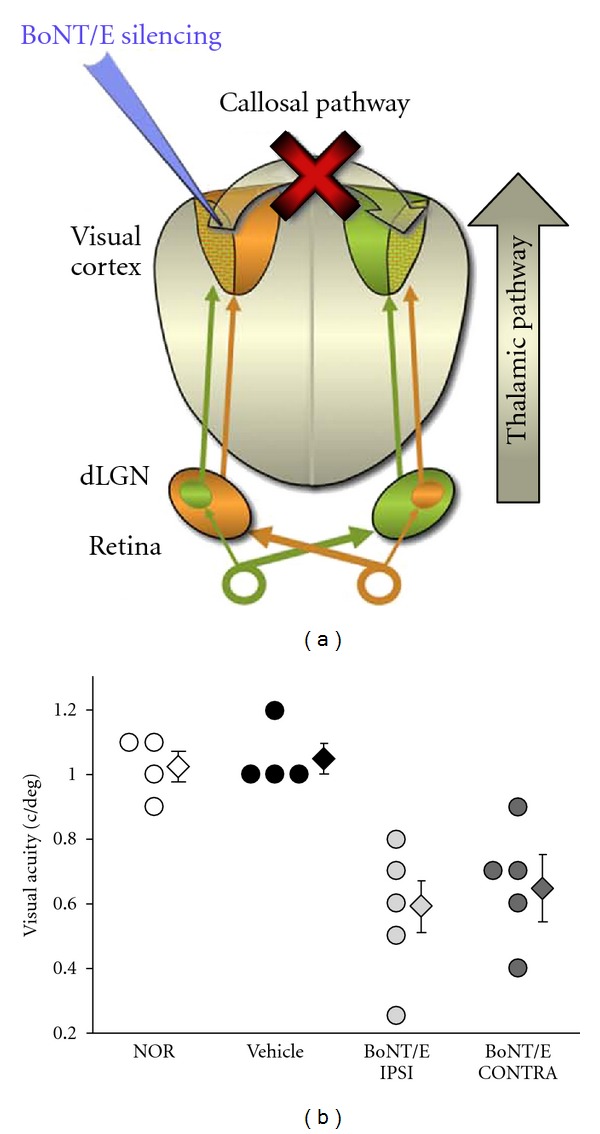
Silencing callosal input during the early critical period impairs maturation of visual acuity. (a) Schematics of the experimental protocol. BoNT/E was unilaterally injected into the visual cortex in P14 rat pups to cause a prolonged silencing of one hemisphere. The contralateral, uninjected side has a normal visual experience through the retinogeniculate pathway and only lacks callosal input activity. (b) Bilateral impairments in visual acuity at P35 after unilateral injection of BoNT/E at P14: summary of visual acuities in naïve rats (NOR), rats injected with vehicle (VEHICLE) and in the hemisphere ipsilateral (IPSI) and contralateral (CONTRA) to BoNT/E infusion. Each circle represents one animal. Mean visual acuity (diamonds) is significantly reduced in both hemispheres of BoNT/E rats in comparison with that in normal or vehicle-injected animals. Error bars indicate SE. Data are from [54].

We unilaterally injected BoNT/E into the visual cortex of rat pups at the time of eye opening [54]. BoNT/E injection resulted in a selective blockade of activity in the injected, but not contralateral, cortex that persisted at least 2 weeks, thus spanning most of the “critical period” for cortical development [71]. This experimental approach is ideal to dissect the role of the interhemispheric connections during cortical development, because the uninjected cortex experiences normal vision through the retinothalamic pathway and only lacks callosal input (see scheme in Figure 1(a)). This transient unilateral silencing of intrinsic cortical activity prevented functional cortical maturation on both sides. The injected cortex displayed deficits in visual acuity and an extension of the critical period for ocular dominance plasticity. Remarkably, these same effects were detectable in the visual cortex of the opposite uninjected side (Figure 1(b)), pointing to a crucial role for inter-hemispheric connections in postnatal development. Thus, maturation of the blocked cortex was superimposable to that of the opposite side, which only lacks callosal input and maintains normal afferent activity through the direct retinogeniculate pathway (Figure 1(b)). The very similar developmental deficits observed ipsilateral and contralateral to the activity blockade indicate a fundamental role for callosal linkages in coordinating the process of cortical maturation [54]. This finding is consistent with the well-known role of the callosum in synchronizing activity in the two halves of the brain [39–42].

Explanations for these bilateral effects after unilateral silencing may implicate the lack of a sustaining callosal input to the opposite visual cortex during activity blockade in one side [54, 77]. In teleological terms, parallel development of the two sides of the brain is needed to ensure a match in information processing between the cerebral hemispheres; the results of these experiments show that transcallosal pathways mediate this coordinated maturation. 

To corroborate this idea, we took advantage of a mouse model with conditional deletion of the AP2*γ* transcription factor. Deletion of AP2*γ* during development results in a specific reduction of upper layer neurons in the occipital cortex, particularly callosally projecting neurons [78]. As a result, adult AP2*γ* conditional knockout mice display reduced size of the corpus callosum. At the functional level, this phenotype was coupled to a profound reduction in visual acuity. As the reduced visual acuity was reminiscent of a physiologically more immature state of the visual cortex, we tested the hypothesis of whether this was also accompanied by maintenance of a higher degree of plasticity, as is the case at more immature stages. Indeed, ocular dominance plasticity triggered by a brief period of MD was retained in adult *AP*2*γ* ^−/−^ mice. These data provide further support for the hypothesis that callosal projections act as an important determinant for the functional maturation of visual cortex [78].

Reports in the literature suggest that the role of the callosum in cortical maturation might be well conserved across species. Indeed, transection of the callosum in kittens during an early phase of postnatal development (but not at later stages) produces a reduction in behaviorally measured visual acuity, supporting a role for interhemispheric communication in cortical maturation [79]. Monkeys that received unilateral lesions of primary visual cortex in infancy display impairments of stimulus detection in the intact visual hemifield [80]. There may be a similar early sensitive period in humans, when callosal integrity appears to be particularly important for the development of visual acuity. Indeed, children born preterm show correlation between white matter microstructure and visual acuity [81]. In keeping with the idea of a “critical period” for the role of the callosum in acuity maturation, adult patients experiencing callosotomy have no impairments of visual acuity [82]. It is important to mention that functional maturation of other cortical properties during development appears to proceed independent of callosal influences. For example, development of orientation selectivity in ferret visual cortex is not affected by activity blockade in the contralateral hemisphere [83].

The development of visual acuity is dependent on proper callosal function only during an early critical period, but the importance of the callosal pathway in integrating cerebral processing is still apparent in adults. Patients with unilateral occipital cortex injury show reduced spatial and temporal sensitivities in the sighted hemifield [84, 85]. Moreover, patients with hemianopia show impairments in figure detection tasks also in the intact hemi-field, suggesting that this deficit may be caused by loss of interhemispheric interactions [86].

## 6. The Role of the Corpus Callosum in Cortical Binocularity

The particularly high concentration of callosal terminals at the area 17/18 border, close to the vertical meridian, prompts for a role of the callosum in binocularity.

In cats, callosal neurons are highly binocular cells (i.e., they respond equally to a stimulus presented to the ipsilateral or the contralateral eye) [7, 87] and a number of experiments have been performed to probe a role for the corpus callosum in eye preference. However, they have yielded contradictory results. Section of the callosum had no effect [88] or lead to a dramatic reduction in binocularity [19, 89–91] in cat visual cortex. The discrepancies in these results may be consequence of technical aspects, including age at which the callosal section is performed and time elapsed between surgery and recording.

**Figure 2:**
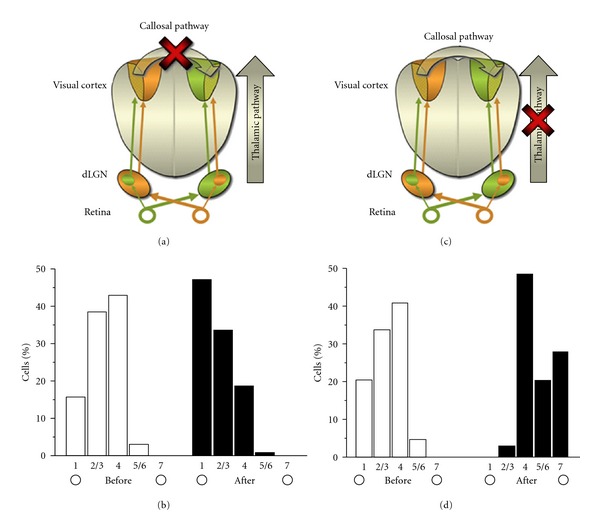
Relative contribution of callosal and thalamic pathways to cortical binocularity. (a, b) Effects of acute callosal silencing on OD in rat primary visual cortex. Binocularity was measured in one side before and after acute pharmacological inactivation of the opposite cortex (a). The results indicate a significant shift of cortical OD towards the contralateral eye, due to the reduction of responses driven by the ipsilateral eye (b). (c, d) Effects of acute geniculate silencing on OD in rat primary visual cortex. The OD histogram shifts towards the ipsilateral eye following acute geniculate inactivation (d). This effect is mainly due to a loss of contralateral eye-driven input. Data are from [32, 55].

In rodents, visual cortex responsiveness is biased towards the contralateral eye [71, 92–94], due to the high percentage (over 95%) of retinal fibres crossing at the chiasm [95], as compared to about 50% of fibers (from the nasal hemiretina) in cats, monkeys, and humans. In rats and mice, the contralateral bias is progressively reduced getting closer to the highly binocular V1/V2 border [92, 96] that maps the vertical meridian and where callosal projections are particularly dense [23, 25]. To clarify the role of the callosum in cortical binocularity in rats, we recorded single unit responses in layers 2-3 before and after acute blockade of callosal input. This was achieved via delivery of muscimol, a GABA_A_ receptor agonist, into the striate cortex contralateral to the recording site (Figure 2(a)). Extracellular recordings of spiking activity demonstrated an enhancement of the contralateral bias of single units following injection of muscimol into the opposite hemisphere [32]. The ocular dominance (OD) shift towards the contralateral eye was due to a reduction of the strength of responses evoked by the ipsilateral eye [32]. The effect was observed for cells with receptive fields close to the vertical meridian and disappeared at more medial locations in the cortex (i.e., within the core of V1, mapping more peripheral parts of the visual hemi-field) [32]. This is consistent with callosal projections being particularly concentrated in the lateral aspect of primary visual cortex (vertical meridian) [23, 25]. Our results are consistent with previous experiments in albino rats that also show a dramatic shift of eye preference (due to loss of ipsilateral eye responses) after cooling of the opposite cortex [97]. 

We have also carried out a complementary experiment where visual responses were recorded before and after acute thalamic inactivation in the same animal. We measured OD before and after removal of the thalamic input with an acute silencing of the geniculate via tetrodotoxin (TTX) injection [55]. This protocol allows one to isolate visual responses driven exclusively by callosal afferents. After geniculate inactivation, OD shifted towards the ipsilateral eye (Figures 2(c) and 2(d)) and this was due to a robust loss of contralateral eye-driven responses, while ipsilateral eye responses were reduced to a less extent [55]. 

Altogether, these data identify two sources of binocularity in rat visual cortex: the retinogeniculate pathway carrying mainly contralateral eye input, and the callosal pathway mainly providing ipsilateral eye input to neurons in the opposite cortex [32, 55, 97]. It is important to mention that the role of callosal input in generating binocular responses might be a specific property of the rodent visual system, given that the proportion of retinogeniculate cortical projections for the ipsilateral eye is low in rodents (due to the massive fiber crossing at the chiasm; see the aforementioned part). Thus, in the normal rat visual cortex, binocularity appears to depend on the function of callosal fibres. In particular, a substantial fraction of the ipsilateral eye drive on cortical responses arrives via callosal connections from the opposite hemisphere, where it is the dominant eye. 

## 7. Role of the Callosum in Ocular Dominance ****Plasticity

Given the importance of callosal input for cortical development and in the generation of binocularity, our group has recently investigated a possible involvement of the callosum in the plastic shift of OD triggered by monocular deprivation. Experience is particularly influential during sensitive periods during development, when appropriate patterns of functional connectivity are selected from wide varieties of potential patterns [98]. During this period, brief modifications of visual experience can induce a profound rearrangement in visual cortical circuitry. Brief MD during the critical period unbalances the amount and pattern of visual information coming from the two eyes and causes an OD shift towards the open eye. In particular, the deprived eye loses its ability in driving cortical neurons (response depression), while neurons respond more vigorously to the open eye (response potentiation).

We deprived a group of rats at the peak of the critical period for seven days to induce the expected shift in OD. Then we measured the OD shift before and after acute silencing of the callosal pathway, via muscimol infusion of the cortex opposite to the recording hemisphere. This experimental protocol allows plasticity to proceed normally and probes the results of acute removal of callosal input. Surprisingly, muscimol injection opposite to the recording cortex restored binocularity after MD in juvenile rats [32]. This recovery of binocularity following callosal silencing was due to an increase in the strength of the deprived eye. Thus, acute removal of callosal influence following MD unmasks deprived eye inputs. These data indicate that callosal afferents act primarily to inhibit closed eye inputs under visual deprivation [32]. In keeping with this observation, continuous silencing of callosal input throughout the MD prevented the loss of responsiveness of the deprived eye, resulting in a dramatic reduction of the OD shift. Thus, transcallosal connections are crucially involved in the weakening of deprived eye responses during MD [32].

An enhanced intracortical inhibition has been previously shown to contribute to the reduced ability of deprived afferents to activate cortical neurons [99]. Our findings demonstrate that callosal inputs are a major source of inhibition following MD. It is likely that this inhibition is relayed by local interneurons, as about 99% of callosal cells are glutamatergic [32]. The specific type(s) of interneurons receiving callosal input are presently not known and are the subject of intense investigation. 

It is interesting to mention that transcallosal inhibition appears to play an important role in plastic events occurring during several brain pathological conditions. For example, in neglect patients some of the behavioural symptoms are attributable to a pathological state of increased inhibition exerted onto the damaged parietal cortex by the contralateral, intact hemisphere [100, 101]. In these patients, silencing the intact side with transcranial magnetic stimulation results in substantial, long-lasting amelioration of the behavioural performances [100, 101].

## 8. Conclusions

In this paper, we have described the contribution of transcallosal pathways to experience-dependent plasticity in the visual cortex. On one side, the fine connectivity of callosal fibers is affected by alterations of visual input (e.g., visual deprivation). On the other hand, the transcallosal route appears to play a critical role in ensuring a functional matching in the developmental maturation of the two cerebral hemispheres during an early critical period. Callosal fibers also contribute to normal binocularity and to the shift of OD occurring after monocular deprivation in rat visual cortex. Thus, the corpus callosum is a key player in the plastic phenomena that underlie adaptation of the juvenile brain to a changing environment.
